# Whole genome sequencing analysis of alpaca suggests *TRPV3* as a candidate gene for the suri phenotype

**DOI:** 10.1186/s12864-024-10086-8

**Published:** 2024-02-16

**Authors:** Stefano Pallotti, Matteo Picciolini, Giovanni Deiana, Dario Pediconi, Marco Antonini, Valerio Napolioni, Carlo Renieri

**Affiliations:** 1 Genomic And Molecular Epidemiology (GAME) Lab, School of Biosciences and Veterinary Medicine, University of Camerino, Via Gentile III Da Varano s/n, 62032 Camerino, Italy; 2 SYNBIOTEC Laboratori s.r.l, Camerino, Italy; 3 School of Pharmacy and Health Products, University of Camerino, Camerino, Italy; 4 Italian National Agency for New Technologies, Energy and Sustainable Development (ENEA), Roma, Italy

**Keywords:** Alpaca, Vicugna, Guanaco, Suri, Huacaya, TRPV3, South American camelids, Fibre production

## Abstract

**Background:**

Alpaca is a domestic South American camelid probably arising from the domestication of two wild camelids, the vicugna and the guanaco. Two phenotypes are described for alpaca, known as huacaya and suri. Huacaya fleece is characterized by compact, soft, and highly crimped fibers, while suri fleece is longer, straight, less crimped, and lustrous. The gene variants determining these phenotypes are still unknown, although previous studies suggested a dominant inheritance of the suri. Based on that, the aim of this study was the identification of the gene variants determining alpaca coat phenotypes through whole genome sequencing (WGS) analysis.

**Results:**

The sample used includes two test-cross alpaca families, suri × huacaya, which produced two offspring, one with the suri phenotype and one with the huacaya phenotype. The analyzed sample was expanded through the addition of WGS data from six vicugnas and six guanacos; this because we assumed the absence of the gene variants linked to the suri phenotype in these wild species. The analysis of gene variant segregation with the suri phenotype, coupled with the filtering of gene variants present in the wild species, disclosed the presence in all the suri samples of a premature termination codon (PTC) in *TRPV3* (transient receptor potential cation channel subfamily V member 3), a gene known to be involved in hair growth and cycling, thermal sensation, cold tolerance and adaptation in several species. Mutations in *TRPV3* were previously associated with the alteration of hair structure leading to an impaired formation of the hair canal and the hair shaft in mouse. This PTC in *TRPV3*, due to a G > T substitution (p.Glu475*), results in a loss of 290 amino acids from the canonical translated protein, plausibly leading to a physiological dysfunction.

**Conclusion:**

The present results suggest that the suri phenotype may arise from a *TRPV3* gene variant which may explain some of the suri features such as its longer hair fibre with lower number of cuticular scales compared to huacaya.

**Supplementary Information:**

The online version contains supplementary material available at 10.1186/s12864-024-10086-8.

## Background

**Fig. 1:**
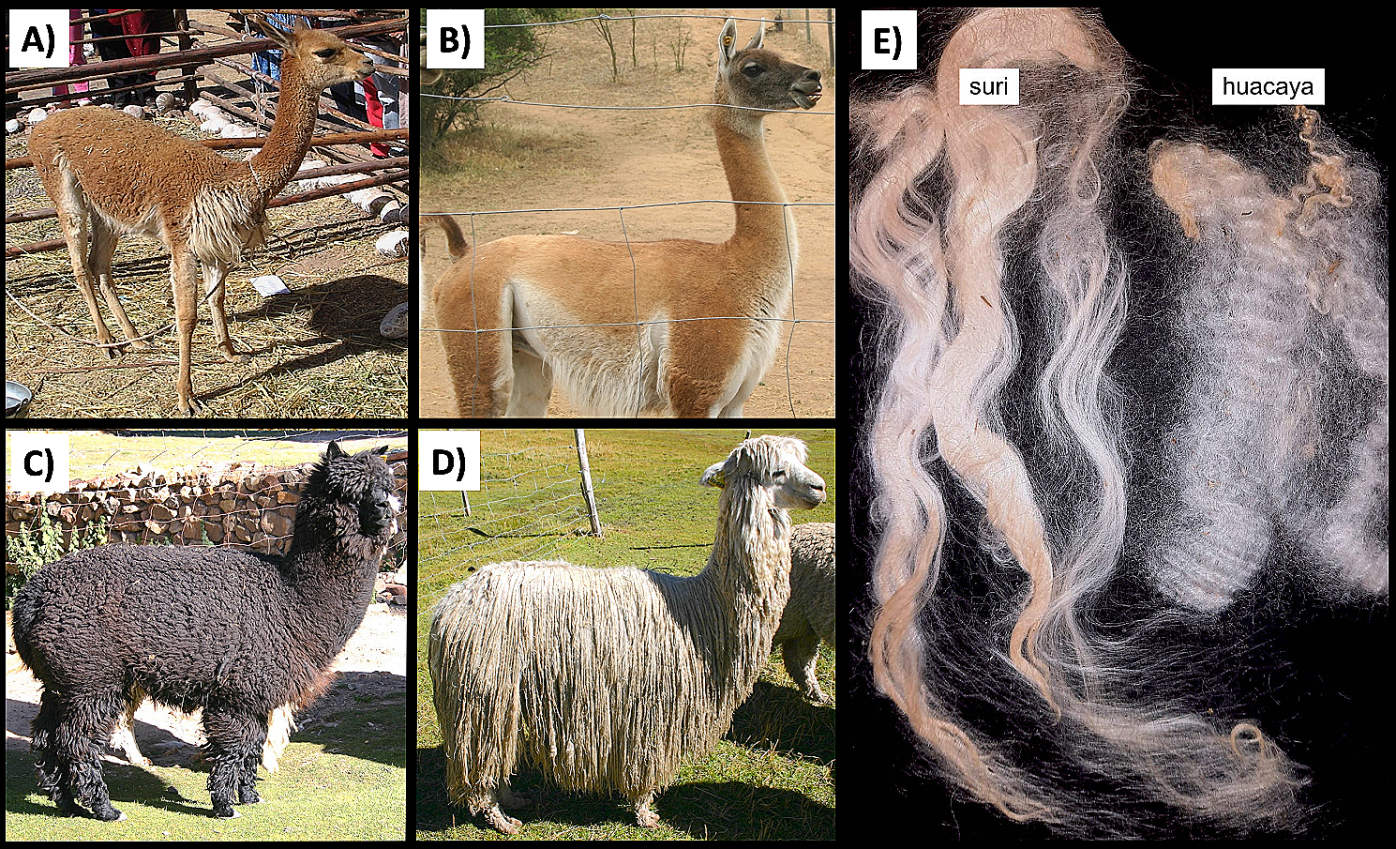
South American camelids. (**A**) Vicugna (*Vicugna vicugna*); (**B**) Guanaco (*Lama guanicoe*); (**C**) Alpaca (*Vicugna pacos*) huacaya; (**D**) Alpaca (*Vicugna pacos*) suri; (**E**) Alpaca fleece

Alpaca (*Vicugna pacos*) is a South American camelid mainly bred in the Andean highlands for its fibre, meat, and transportation [1, 2]. This species is believed to arise from the domestication of the wild camelids, the vicugna (*Vicugna vicugna*) (Fig. 1A) [3] and the guanaco (*Lama guanicoe*) (Fig. 1B) [4] or as hybrid between the vicugna and the domestic llama (*Lama glama*) [5, 6]. However, the real ancestry of alpaca is still not completely understood, and both vicugna and guanaco may have contributed to the evolution of this domestic species.

Based on the coat phenotype, two different variety are described for alpacas known as huacaya, the most common (Fig. 1C) [7] and suri (Fig. 1D), which encompasses about the 7% of the entire South American alpaca population [8]. Therefore, huacaya is often reported as the wild-type alpaca selected from the double coated vicugna for domestication while suri is thought to be derived from huacaya through gene mutation with reduction of fitness [7, 9] and increased delicacy in harsh climatic conditions as frequently reported by Andean breeders [10]. The longer hair shaft observed for the suri alpaca is the main different feature compared to huacaya [11, 12]; however, the two phenotypes differ in other coat characteristics and in microscopic qualities of the fibre. In fact, while the huacaya fleece is characterized by compact, soft, and highly crimped fibers, the longer suri fleece is straight, less crimped and lustrous (Fig. 1E) [13]. Moreover, the suri fibre also differs for the lower number of cuticular scales respect to huacaya (and llama) [14, 15].

Different works tried to unveil the genetic variants determining these two phenotypes. Segregation studies suggested that two linked loci must simultaneously be homozygous for recessive alleles to produce the huacaya phenotype while the suri phenotype is determined by the presence of a dominant allele at either locus [7]. More recently, a premature termination codon (PTC) due to C > T substitution (p.Arg167*) was identified in *FGF5* (fibroblast growth factor 5), a gene involved in the elongation of the fibre [13], and genome-wide studies found selection signals on this gene [5, 6]. Nevertheless, such variant was observed in both phenotypes and, although it may explain the coat differences observed between alpaca and its wild ancestor, it cannot account alone for the differences observed between suri and huacaya coats. In this regard, other unknown genetic variants may explain such phenotype variation.

The advent of next-generation sequencing led to an enormous amount of freely available whole genome sequencing (WGS) data from several species [16], which proved to be helpful in the evolutionary and zootechnical research on South American camelids [5, 6]. A comprehensive WGS analysis encompassing domestic alpacas and its wild ancestors, vicugna and guanaco, would improve the identification of the genetic variants linked to fibre production in the different domestic camelid species. However, no study so far has leveraged a WGS approach to understand the gene variant involved in the development of the alpaca coat features. Thus, the aim of this study was to identify the gene variants responsible of the suri and the huacaya phenotypes, through a WGS analysis. By performing a joint variant calling between suri alpaca, huacaya alpaca, vicugna and guanacos, we identified a premature termination codon (PTC) on the *TRPV3* (transient receptor potential cation channel subfamily V member 3) gene, segregating with the suri phenotype which may explain some of the features of its fibre.

## Results

### Identification of the gene variants linked to alpaca suri phenotype

The genomic joint variant calling was performed on a final sample encompassing three huacaya alpacas, three suri alpacas, six wild vicunas and six wild guanacos. A total of 47,542,580 variants were called of which 39,944,120 were classified as single nucleotide variants (SNVs), while 3,911,560 and 3,686,900 were classified as insertions and deletions, respectively. After the variant annotation process, 298,362 were classified as missense variants, 3,284 as nonsense variants and 461,292 as silent variants.

Assuming a dominant inheritance model for the suri phenotype, nonsense and missense variants were further analyzed to filter the heterozygous variants present only in the three suri samples using the huacaya alpacas, vicugnas and guanacos as control samples. The analysis revealed eight variants with potential high phenotypic impact located on eight loci (Table 1). Frameshift variants were identified in the uncharacterized *LOC116280269*, *DNAJC22* (DnaJ heat shock protein family member C22), *TRNAT-AGU45* (transfer RNA threonine (anticodon AGU)) and *PLIN5* (perilipin 5) located on chromosomes 4, 12, 13 and 22, respectively. A start-lost codon was identified for *LOC102534084* (TRAV13-1: T cell receptor alpha variable 13-1-like) located on chromosome 6, while a premature termination codon (PTC) was identified in *TRPV3* (transient receptor potential cation channel subfamily V member 3) located on chromosome 16, in *ARPC4* (actin related protein 2/3 complex subunit 4) located on chromosome 17, and in *HELZ2* (helicase with zinc finger 2) on chromosome 19. Additionally, 153 missense variants located on one 136 different loci were found (Table S1).

**Table 1:** High impact variants found in alpaca suri

Locus	Chromosome	Alpaca huacaya, vicugna and guanaco genotype	Alpaca suri genotype	Genomic position of the variant in alpaca suri	Predicted phenotypic effect of the variant in suri	Effect T ype of the variant
uncharacterized LOC116280269	4	*CA/CA*	*CA/C*	c.717delA	p.Lys239fs	frameshift variant
LOC102534084 (TRAV13-1)	6	*A/A*	*A/T*	c.2T > A	p.Met1?	start lost
DNAJC22	12	*G/G*	*G/GT*	c.72_73insA	p.Leu25fs	frameshift variant
TRNAT-AGU_45	13	*C/C*	*C/CCCCG*	c.60_63dupCCCG	p.Val22fs	frameshift variant
TRPV3	16	*G/G*	*G/T*	c.1423G > T	p.Glu475*	stop gained
ARPC4	17	*G/G*	*G/A*	c.301 C > T	p.Gln101*	stop gained
HELZ2	19	*C/C*	*C/A*	c.4320 C > A	p.Cys1440*	stop gained
PLIN5	22	*G/G*	*G/GT*	c.912dupT	p.Gly305fs	frameshift variant

A comprehensive literature search was performed to find scientific evidence about any relation between the filtered variants and the hair follicle biology.

Based on bibliographic sources, *TRPV3* resulted the most promising candidate gene potentially involved in the suri phenotype due to its strong correlation with the hair follicle biology, therefore this gene was considered for further analysis.

It must be noted, however, that ten out of 136 loci harboring a missense variant (i.e.: *RYR3, CADM1, NBN, PTK6, MYO5A, USP25, RNF175, ABCA4, CD44* and *RASAL2*), are reported in scientific literature as expressed in the hair follicle, keratinocytes or related to coat phenotypes. Nevertheless, the limited number of papers available for such loci did not allow to speculate about any relation with the suri coat phenotype and more studies are needed to clarify their association to alpaca coat features. Information on these genes as well as the bibliographical references are reported in table S1.

### Protein functional domains prediction and modeling

**Fig. 2:**
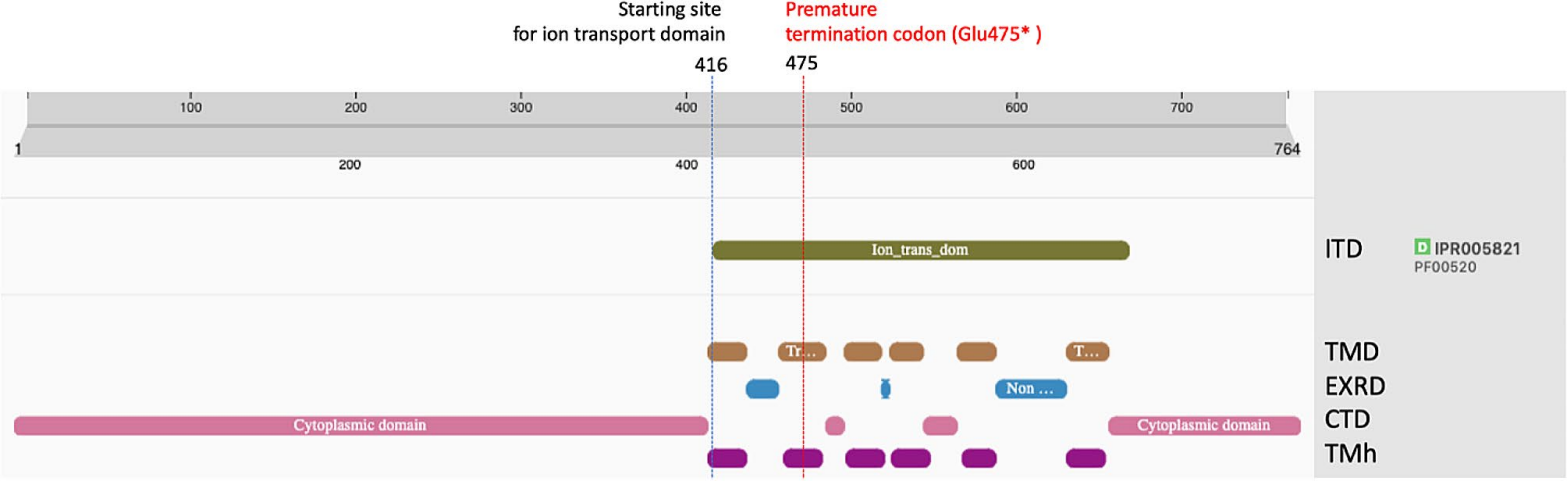
Protein functional domains prediction for alpaca TRPV3. ITD = Ion transport domain; TMD = Region of a membrane-bound protein predicted to be embedded in the membrane; EXRD = Region of a membrane-bound protein predicted to be outside the membrane, in the extracellular region; CTD = Region of a membrane-bound protein predicted to be outside the membrane, in the cytoplasm; TMh = Transmembrane helices

**Fig. 3:**
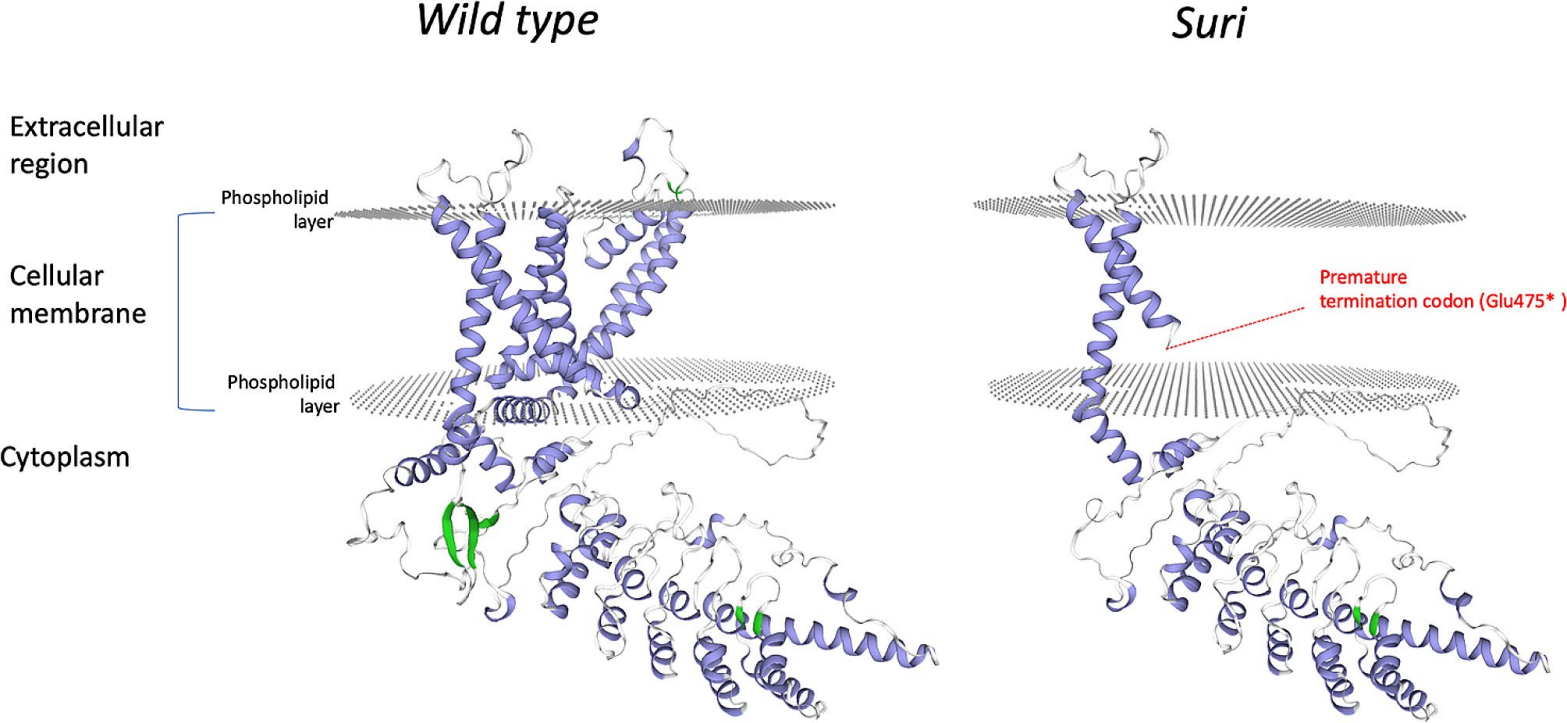
Protein models for alpaca TRPV3 in huacaya (wild type) and suri

The prediction of the TRPV3 functional domains in alpaca revealed an ion transport domain starting from aminoacidic position 416 encompassing six transmembrane helices, three extracellular regions and three cytoplasmic regions (Fig. 2). The wild-type protein model built with SWISS-MODEL had a GMQE score of 0.75, a sequence identity of 89.5% and a coverage score of 1.00 (amino acidic range 118–731) while the suri mutated protein model built had a GMQE score of 0.73, a sequence identity of 85.7% and a coverage score of 1.00 (amino acidic range 85–473). The analysis of the suri mutated TRPV3 protein showed that the premature termination codon (p. Glu475*) occurred in the second transmembrane helices of the protein leading to the putative loss of the whole downstream amino acidic chain (Fig. 3).

## Discussion

Assuming the suri phenotype as dominant trait, our work aimed to identify the suri variants segregating in two alpaca families using huacaya and the two wild ancestors, vicugna and guanaco, as control. WGS of alpaca are available from previous studies [6]; however, the real phenotype related to the hair coat type is often unclear, thus we expanded our sample by adding data from twelve alpaca’s wild ancestors (six vicugna and six guanaco) for which the phenotype related to the hair coat is known. We found heterozygote genotypes at variants of high impact in eight loci along with missense mutations in 136 loci in all the suri samples (Table 1, Table S1). After performing a comprehensive scientific literature search, the most promising candidate variant was the PTC due to G > T substitution (p.Glu475*) in *TRPV3*, a gene belonging to a superfamily with more than 30 members in mammals [17].

TRPV3 is a tetramer composed of six transmembrane (TM) domains and two cytoplasmic amino- and carboxy- termini. The central TM domains are evolutionarily conserved among vertebrates with the pore loop for ion flux located between TM5 and TM6 [17]. The gene is highly expressed in keratinocytes, inner root sheath and the hair shaft working as a thermosensitive TRP channel [17]. *TRPV3* acts as key regulator of epidermal development, hair growth and cycling [18, 19] working as catagen activator by the inhibition of hair shaft elongation and hair matrix keratinocyte proliferation, inducing apoptosis-driven premature catagen regression [18]. Moreover, in several mammal species, *TRPV3* is a key gene for the normal fetal hair follicle development [20, 21], thermal sensation, cold tolerance, and adaptation [22–27]. The hair follicle cycle, a process characterized by cyclic phases of growth (anagen), involution (catagen) and quiescence (telogen) of hair follicles, is altered in *TRPV3*-mutant mice [17, 28]. Indeed, gain-of-function mutations alter hair structure, leading to an impaired formation of the hair canal and the hair shaft. Additionally, *TRPV3* mutant mice exhibit increased proliferation in the outer root sheath, accelerated hair cycle, reduction of hair follicle stem cells, miniaturization of hair follicles and reduction of hair diameter [29, 30]. Mutations also cause hair loss in hairless *TRPV3* mutant rodent strains [31], with a phenotype that is inherited in an autosomal dominant fashion [32]. On the other side, *TRPV3* knockout mice exhibit alteration on hair development characterized by wavy hair coat and curly whiskers [33].

*TRPV3* acts as a regulator of the catagen phase also in human. In organ-cultured hair follicles the expression of this gene induces an apoptosis-driven premature catagen regression through the inhibition of hair shaft elongation and hair matrix keratinocyte proliferation [18, 19]. The same effect can be induced through the activation of *TRPV3* with chemical agonist compounds. On the contrary, the chemical inhibition of *TRPV3* reverses the hair growth suppression [reviewed in 28]. *TRPV3* mutations were also suggested to drive hair loss in patients with Olmsted syndrome (OMIM#614,594) [reviewed in 28].

*TRPV3* influences the development of skin and hair coat features also in other mammals. Indeed, the mutation of *TRPV3* was reported as positively selected in the stem lineage of woolly mammoths suggesting a contribution to evolution of cold tolerance, long hair, and large adipose stores [26, 27]. Wu et al. [34] identified *TRPV3* mutations in ruminants living at high altitudes such as sheep (*Ovis aries*) and bighorn sheep (*Ovis canadensis*), suggesting a link between the gene and the wool development which may provide better protection against the cold than the normal skin. Mutations on *TRPV3* were found also in the American beaver (*Castor canadensis*), potentially associated with their scaly stratum corneum in the tail, and in species inhabited hot stressful environments as the sunda flying lemur (*Galeopterus variegatus*), lesser Egyptian jerboa (*Jaculus jaculus*), and wild Bactrian camel (*Camelus ferus*). The authors suggested that such mutations might be related to the tolerance against high temperatures because of the thermosensation function of the gene.

Based on these previous studies, the *TRPV3* variant found in our study, suggests a link with the suri phenotype due to the key role of the gene in the hair follicle biology. In our results, all the suri samples showed a premature termination codon due to G > T substitution (p.Glu475*) leading to the potential loss of 290 amino acids from the translated TRPV3 protein (Figs. 2 and 3) and consequent possible disruption of its normal function. As shown in Table 2, this variant segregated in the two alpaca families in an autosomal dominant fashion as previously proposed by Presciuttini et al. (2010) [7]. The protein modeling and functional domains prediction suggested a strong deficiency in the structure of the TRPV3 mutated protein. In the suri sample in fact, the premature termination codon observed in position 475 (Glu475*) is in the second transmembrane domain (Figs. 2 and 3) leading to a shorter TRPV3 protein lacking five transmembrane domains along with the pore loop essential for the ion transport. Thus, the normal function of the protein would be impaired. Considering the essential role of the TRPV3 in triggering the hair follicle catagen, the mutation may potentially cause a delay in the onset of this phase resulting in a persistence of anagen stage and consequent increase in the hair shaft length which characterizes the suri phenotype [11, 12].

**Table 2:** Segregation of TRPV3 c.1423G > T (p.Glu475*) variant in alpacas families

Family	Phenotype	Genotype
	Suri (Dam)	*G/T*
1	Huacaya (Sire)	*G/G*
	Suri (Kid)	*G/T*
	Huacaya (Dam)	*G/G*
2	Suri (Sire)	*G/T*
	Huacaya (Kid)	*G/G*

Other fibre characteristics observed in suri suggest a potential role of *TRPV3* Glu475* in the development of its peculiar phenotype. In fact, suri fibre clearly differentiates from those of other camelids by showing a lower number of cuticular scales compared to huacaya alpaca and llama [14, 15]. Such features may arise from the *TRPV3* variant which alters the hair follicle inner root sheath and keratinocytes where the gene is highly expressed leading to an impaired formation of the hair shaft [17, 30]. It must be noted that *TRPV3* has a pleiotropic effect as the TRP channels which play an important role in the regulation of various cell functions. In fact, *TRPV3* mutations associated with a variety of integumentary diseases such as Olmsted’s syndrome [29, 30] while increased gene expression was observed in several types of cancer and cardiac diseases [35]. On this respect the variant *TRPV3* Glu475* may suggest the hypothesis that the suri phenotype is autosomal dominant and the mutation is most likely homozygous embryonic lethal as proposed for other alpaca phenotypes as the classic gray coat [36]. In this regard, it must be stressed the low frequency for the suri phenotype [8] which suggests a reduction of fitness (Escobar, 1984) [9]. Moreover, the function of the TRPV3 in the regulation of thermal adaptation and cold tolerance could be impaired in suri; in fact, there are casual reports of breeders on higher weakness of this phenotype as regards growth, diseases or mortality, and it has been hypothesized the suri hair coat type might have fewer protective properties against the extreme Andean climatic conditions [10].

### Limitations

The present study presents some limitations which must be discussed. First, although our experimental design allowed to identify the variants segregating with the suri alpaca phenotype, the results must be validated on a larger sample. Furthermore, the potential impairment of the TRPV3 protein in suri suggested by our results should be further confirmed throughout transcriptomic and proteomic approaches. Others analysis, such as the estimation of the age of a genetic mutation [37], may be applied to further understand the presumed selection of the suri phenotype from huacaya alpaca; however, these methods require recombination maps which are still not available for such species.

## Conclusion

Previous studies reported the central role of the *TRPV3* in the biology of hair follicle, hair structure and thermal adaptation. These biological activities are impaired when mutations in this gene occur. Taken together, our results correlate with the hypothesis that the suri phenotype may arise from a mutation on *TRPV3* gene. The finding of a premature termination codon on this pleiotropic gene in fact, may explain some of the suri features such as its longer hair fibre with lower number of cuticular scales compared to huacaya, along with its autosomal dominant inheritance with potential reduction of fitness. Other studies are required to understand the impact of the *TRPV3* mutation in alpaca biology; however, our work provides a further advancement in the understanding of the gene variants behind the suri phenotype.

## Methods

### Sample collection

The six alpacas used for the study belonged to two test-cross pairs: suri female × huacaya male and huacaya female × suri male which gave birth to one cria with suri phenotype and one cria with huacaya phenotype, respectively (Table 3). The animals were raised at the experimental station of the INIA (the Peruvian National Institute for Agronomic Innovation) located in Quimsachata, Peru [38]. The four parents chosen belonged to pure-line animal populations (suri x suri and huacaya x huacaya) selected for twenty years (with a generation interval of about 4 to 5 years) [39].

**Table 3:** WGS sample description

Species-Breed	Batch	SRA id	Sequence length	Coverage	Genotyping rate
Alpaca - Suri (Dam)	Family 1; In house sample	-	150	62	100
Alpaca - Huacaya (Sire)	Family 1; In house sample	-	150	42	99.9
Alpaca - Suri (Offspring)	Family 1; In house sample	-	150	50	99.9
Alpaca - Huacaya (Dam)	Family 2; In house sample	-	150	37	99.9
Alpaca - Suri (Sire)	Family 2; In house sample	-	150	51	99.8
Alpaca - Huacaya (Offspring)	Family 2; In house sample	-	150	43	99.8
Guanaco	PRJNA612032	SRR11905252	150	15	93.9
Guanaco	PRJNA612032	SRR11905249	150	20	99.5
Guanaco	PRJNA612032	SRR11905250	150	19	99.5
Guanaco	PRJNA612032	SRR11905251	150	19	99.5
Guanaco	PRJNA612032	SRR11905261	150	18	99.6
Guanaco	PRJNA612032	SRR11905272	150	18	99.5
Vicugna	PRJNA612032	SRR11905260	150	21	99.7
Vicugna	PRJNA612032	SRR11905262	150	20	99.7
Vicugna	PRJNA612032	SRR11905264	150	18	99.6
Vicugna	PRJNA612032	SRR11905265	150	16	99.6
Vicugna	PRJNA612032	SRR11905266	150	16	99.6
Vicugna	PRJNA612032	SRR11905267	150	18	99.6

Skin biopsies were performed as described in Pallotti et al., [13] and were used for the *de novo* sequencing. Genomic DNA was isolated using the Genomic DNA Isolation Kit (Norgen Biotek Corp.), according to the manufacturer’s instructions. The library preparation was carried out at Genomix4Life (Salerno, Italy) using the Illumina DNA Prep Kit (Illumina) followed by a 150 bp sequencing at paired-end mode, using the Illumina NovaSeq 6000 System.

The analyzed sample was further expanded by adding six vicugna and six guanaco WGS samples generated by a previous project (PRJNA612032) retrieved from the NCBI Sequence Read Archive (SRA) (Table 3). The twelve wild animals were added as control samples to the dataset, assuming the absence in their genome of the variants linked to the typical suri fiber. The public SRA files were downloaded to our server and converted to FASTQ files. The final sample used for the analysis encompassed three huacaya alpacas, three suri alpacas, six wild vicugnas and six wild guanacos (Table 3).

### WGS quality control and variant calling

The quality of the FASTQ files was checked using FastQC [40] and the adapter trimming was performed with Trimmomatic [41]. Read pairs were mapped to the alpaca reference genome *VicPac3.1* [42] using Burrows-Wheeler Alignment MEM (BWA-MEM) [43]. The samples showed a genotyping rate higher than 99% (excepting for one guanaco sample [SRR11905252]), with a sequencing depth coverage rate ranging from 15 to 63X (Table 3). BAM files were further processed using the Genome Analysis Toolkit (GATK, v3.4) [44] and the HaplotypeCaller approach was used for variant calling [45].

### Annotation of the variants and filtering

The VCF containing the genomic variant calling of the 18 samples was annotated using SNPeff [46]. Finally, the heterozygous variants segregating in the three suri samples were filtered using SNPsift [47].

### Selection of the variants

An extensive literature search was carried out on PubMed and Google free search engines for the genes harboring start-lost codon, frameshift and missense mutations using the keywords: “gene name” and “hair” applying the following algorithm: (gene name) AND (hair).

### Assessments of the fidelity of variants

**Fig. 4:**
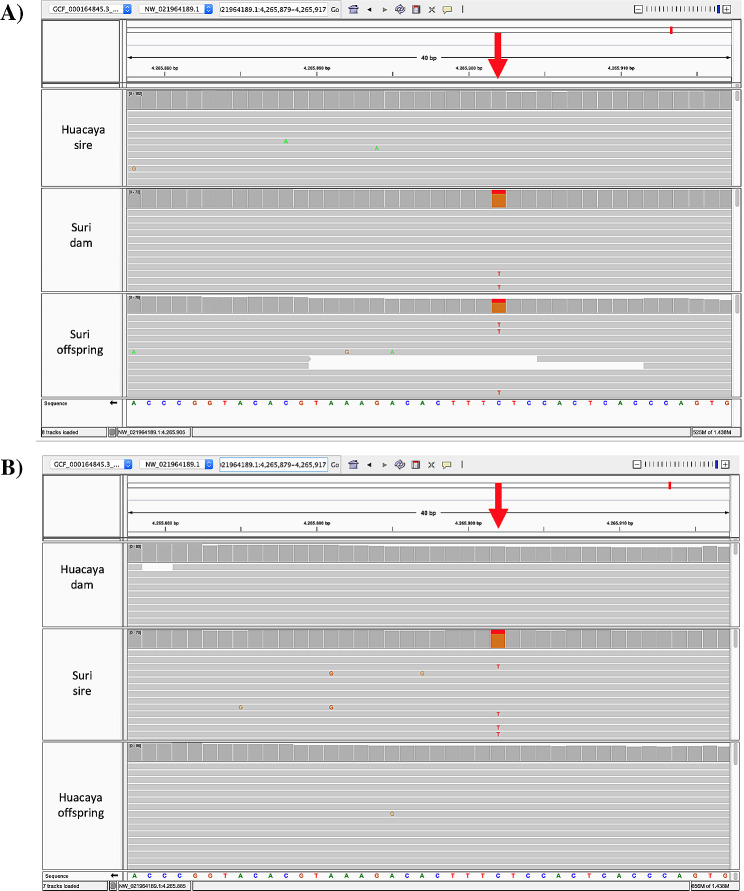
Alignment of BAM files for the manual inspection of the G > T substitution (p.Glu475*) in TRPV3 in the Integrative Genomics Viewer. Red arrows indicate the position of the variant. (A) Alpaca family #1; (B) Alpaca family #2

The resulting read alignments from BAM files were visualized in the Integrative Genomics Viewer and manually inspected to verify the fidelity of variants [48] (Fig. 4).

### Protein functional domains prediction and modeling

The functional domains of the TRPV3 protein were predicted using InterProScan [49]. The structural protein model was built using the SWISS-MODEL workspace [50] using both human and mouse TRPV3 protein sequence as template due to the high score reported by the software for the Global Model Quality Estimate (GMQE) (a quality estimate combining properties from the target-template alignment and the template structure) the sequence identity, and the coverage.

### Electronic supplementary material

Below is the link to the electronic supplementary material.

Supplementary Material 1

## Data Availability

The VCF file from the *de novo* WGS of six alpacas used in the current study are available in the EVA (European Variation Archive) repository under the project PRJEB61878 [https://www.ebi.ac.uk/eva/?eva-study=PRJEB61878] while the FASTQ files are available in the NCBI SRA (Sequence Read Archive) repository under the project no. PRJNA1020284. The six vicugna and the six guanaco samples generated by a previous project and used in this study are available in the NCBI Sequence Read Archive (SRA) [PRJNA612032].

## Abbreviations

WGS: Whole-genome sequencing
TRPV3: Transient receptor potential cation channel subfamily V member 3
PTC: Premature termination codon
